# Climatic similarity and biological exchange in the worldwide airline transportation network

**DOI:** 10.1098/rspb.2007.0148

**Published:** 2007-04-10

**Authors:** Andrew J Tatem, Simon I Hay

**Affiliations:** 1 Spatial Ecology and Epidemiology Group, Tinbergen Building, Department of Zoology, University of OxfordSouth Parks Road, Oxford OX1 3PS, UK; 2 Malaria Public Health and Epidemiology Group, Centre for Geographic Medicine, KEMRI/Wellcome Trust Research LaboratoriesPO Box 43640, 00100 Nairobi, Kenya

**Keywords:** air travel, biological invasion, network analysis

## Abstract

Recent increases in the rates of biological invasion and spread of infectious diseases have been linked to the continued expansion of the worldwide airline transportation network (WAN). Here, the global structure of the WAN is analysed in terms of climatic similarity to illuminate the risk of deliberate or accidental movements of climatically sensitive organisms around the world. From over 44 000 flight routes, we show, for each month of an average year, (i) those scheduled routes that link the most spatially distant but climatically similar airports, (ii) the climatically best-connected airports, and (iii) clusters of airports with similar climatic features. The way in which traffic volumes alter these findings is also examined. Climatic similarity across the WAN is skewed (most geographically close airports are climatically similar) but heavy-tailed (there are considerable numbers of geographically distant but climatically similar airports), with climate similarity highest in the June–August period, matching the annual peak in air traffic. Climatically matched, geographically distant airports form subnetworks within the WAN that change throughout the year. Further, the incorporation of passenger and freight traffic data highlight at greater risk of invasion those airports that are climatically well connected by numerous high capacity routes.

## 1. Introduction

Throughout recent history, the geographical isolation between plants and animals has been gradually eroded by the deliberate or accidental transport of organisms caused by human travel, tourism or trade. Today, the rate at which species are moving between different biogeographic regions is unprecedented (Mack *et al*. 2000), resulting in adverse ecological (Gewin 2005), economic (Pimentel *et al*. 2000) and human health consequences (Soper & Wilson 1943; Lounibos 2002; Tatem *et al*. 2006*c*). Reducing these problems requires examination of the ways that humans facilitate the transport and establishment of organisms in new areas (Floerl & Inglis 2005).

Biological invasion is a multi-step process comprising initial dispersal, establishment and spread (Elton 1958; Shigesada & Kawasaki 1997). During each step, selective pressures act on the survival of organisms to decrease successively the overall pool of species and probability of invasion success (Williamson 1996). While substantial research and resources have been directed towards the final steps in documenting the spread of invasions, and the characterization and identification of potentially invasive species or vulnerable ecosystems (Kolar & Lodge 2001; Gewin 2005), the actual routes over which climate-sensitive organisms might initially be dispersed, and survive after arrival, have received relatively little attention (Puth & Post 2005).

International transport networks and hubs are particularly important in providing movement routes and gateways into new regions for the spread of exotic species (Drake & Lodge 2004; Tatem *et al*. 2006*c*). How often a species is transported and how many individuals survive (‘propagule pressure’), as well as the simple establishment of a route are thought to be important correlates of establishment success (Levine & D'Antonio 2003; Lockwood *et al*. 2005). An increasingly important contributor to the international movement of organisms is the worldwide airline transportation network (WAN) which, in terms of passengers carried, has expanded at a rate of about 8% per annum for the last 3 years, with projections forecasting this to continue for at least the next 5 years (IATA 2006).

International air travel has been recently pinpointed as a significant factor in the movement of economically damaging pest species, with 73% of pest interceptions in the US Port Information Network database occurring at international airports (Liebhold *et al*. 2006; McCullough *et al*. 2006). Among others, the Mediterranean fruit fly (*Ceratitis capitata*) has been consistently imported in airline baggage (Liebhold *et al*. 2006), plant pathogens are often found in air cargo (McCullough *et al*. 2006) and disease-carrying mosquitoes have survived long haul flights in aircraft cabins (Lounibos 2002; Tatem *et al*. 2006*b*). Despite this, analyses on the potential role of the WAN in biological invasions remain few (Tatem *et al*. 2006*a*–*c*). With the WAN expanding, continued trade liberalization and limited funds available for surveillance and control, multidisciplinary approaches are required for the identification of both routes and times of year when the long distance movement of organisms is most likely to occur. Liebhold *et al*. (2006) showed that the number of alien insect species interceptions at airports across the US was positively related to the incoming air traffic volumes. Tatem *et al* (2006*a*,*c*) went one step further and showed that the international spread of the Asian tiger mosquito, *Aedes albopictus*, could be explained using data on both traffic volumes between source and potential invasion points, and the climatic similarity between them; neither category of information alone could explain as much of the historical pattern of spread as both combined. Here, the same approach is applied on a seasonal basis to the WAN, to reveal how seasonal variation in invasion risk might be related to passenger volumes (a surrogate for propagule pressure, Levine & D'Antonio 2003; Drake & Lodge 2004; Lockwood *et al*. 2005) and climatic linkages (favouring organism survival after arrival). The analysis is generic, and not restricted to any particular species, but is likely to be most relevant to those microorganisms, plants and insects that are highly sensitive to small climatic changes. This particularly includes the important group of insect pests and disease vectors that are both more active and abundant in warmer (‘summer’) conditions.

Seasonal levels of overall climatic similarity within the WAN are explored, as well as how best to relate these to typical traffic volumes. The scheduled routes within the WAN that connect spatially distant, but climatically similar airports, and the airports with numerous incoming routes of this type (potential invasion ‘hot spots’) are then identified month by month for a typical year. Finally, significant subnetworks within the WAN that connect airports with similar climatic regimes are mapped, and the way that traffic volumes affect findings are examined.

## 2. Material and methods

### (a) Worldwide airline transportation network data

Flight schedule data of over 800 of the world's airlines for the 12-month period of 1 May, 2005 to 30 April, 2006 were obtained from the OAGMAX database (http://www.oagdata.com/solutions/max.aspx). This database is compiled by OAG Worldwide (Downers Grove, IL), and includes all scheduled passenger, charter and freight flights, both for large (air carriers) and small aircraft (air taxis). During the period considered, there were 3 219 774 scheduled flights operating between 3570 airports on 44 285 routes (stopovers not included). Twelve traffic capacity matrices for the WAN were created, one for each month, with each cell containing the total seat capacity between each airport. Though the number of seats does not necessarily equate to number of passengers, comprehensive data on origin–destination passenger numbers on individual routes do not exist, therefore, seat capacity was used as a surrogate. Strong year-round correspondence between total seat capacity, hold volume and aircraft tonnage indicated this to be an adequate measure of traffic (passengers and freight) within the WAN (figure 1 in the electronic supplementary material).

### (b) Climatic distances

A 10′×10′ (approx. 18×18 km at the equator) spatial resolution gridded climatology (New *et al*. 2002) was used to create mean temperature, rainfall and humidity surfaces for the 12 months of a synoptic year. These surfaces were linearly rescaled to the common data range, 0–1, and the locations of the airports were superimposed onto them. For every month of the year, each grid square covering the location of each airport was identified. To ensure a representative climate measure, up to eight land pixels surrounding each airport grid square were identified wherever possible. Any airport located on islands too small to be represented by the climate surfaces was eliminated from the analysis, reducing the sample size to 3364 airports. The remaining network included 99.96% of the global seat capacity from the original database. For each month, the selected grid square data from the climate surfaces thus formed the climatic ‘signature’ of each airport.

The climatic similarity of a scheduled air route connecting airport *i* with airport *j* in the WAN is defined here by the Euclidean distance between the climatic conditions at *i* and those at *j*. A relatively high climatic Euclidean distance (CED) on a route shows that the regions connected are climatically dissimilar, while a relatively low CED indicates climatic similarity. The Euclidean distance in climatic space between each airport signature and every other airport signature was calculated to produce 12 symmetrical airport ‘climatic dissimilarity’ matrices, one for each month of the year, with each cell representing a CED between one airport and the other. Euclidean distance measures were used because there were generally very few pixels for any more sophisticated measures of environmental distance. Moreover, in the few cases where they could be applied, more complex measures of distance did not affect the results. Finally, CEDs between airports where no scheduled route exists in the WAN were removed from the dissimilarity matrices.

### (c) Subnetworks

The entire WAN, with routes weighted by CEDs, produces a proportional strength network (a network where each connecting line has a value, or ‘strength’) with a foundation for detecting sets of strongly related airports climatically (subnetworks). Identification of such subnetworks groups those airports and regions between which biota exchange is more likely, and identifies from which areas and times of year airports can expect an increased risk of incoming, potentially successful biological invaders.

The identification of subnetworks was undertaken through the mapping of simple ‘line islands’ (Batagelj & Mrvar 1998, 2002; Batagelj & Mrvar 2003; de Nooy *et al*. 2005). Line islands are connected subnetworks (groups of airports with connecting flight routes) identified by the magnitude of the CED of its routes (line weights). The approach enables the identification of distinct subnetworks in weighted networks, and produced very similar groupings to those indicated by subjective cut-offs through hierarchical clustering. The subnetworks of interest here were those that encompassed routes with exceptionally low CEDs, indicating well-connected groups of airports with similar climates in the month in question. To meet the algorithm requirements, before the calculation of subnetworks, the CEDs were inverted by calculating their reciprocals, ensuring that the most climatically similar airports were connected by the largest line weights. A set of airports forms a line island within the WAN if, and only if, this subnetwork induces a connected subgraph (all airports are connected to at least one other in the subnetwork by scheduled flight routes) and the CEDs on the connecting routes are lower and more strongly related among the airports making up the subnetwork than with other, directly connected airports in the WAN.

Month by month, those subnetworks within the WAN of size 2<*n*<1000 airports were identified. Throughout the year, the largest island with the strongest line weights consistently encompassed northern Europe and eastern USA. This was subdivided to identify important groupings of climatically similar airports within it, by adjusting the thresholding to 2<*n*<500 airports.

### (d) Traffic

Colizza *et al*. (2006) showed that the WAN is highly heterogeneous in terms of traffic capacities and the traffic volume distribution is skewed and heavy tailed, with the highest capacity routes often being the shortest ones. Numerous long distance routes of similar capacities are also in operation, however, and it is these that are of interest in terms of biological invasion, especially those where CED is low. To examine the general combined effect of climatic similarity and traffic volume for each route and month, traffic-scaled climatic Euclidean distance, CED*t* was calculated. The traffic-scaled CED between airports *i* and *j*, CED*t*_*ij*_, is defined as

$${\text{CED}t}_{ij}=\left(\frac{1}{{\text{CED}}_{ij}}\right)t_{ij},$$

where CED_*ij*_ is the CED between airports *i* and *j*, and *t*_*ij*_ is the total monthly seat capacity on the route from airport *i* to airport *j*.

For a scheduled flight route, a low CED*t* indicates that, for the month in question, the traffic volume and climatic similarity between the connected regions is low. Conversely, a high CED*t* on a route shows there to be high levels of traffic between climatically similar regions. This case represents a greater possibility of biological invasion, as greater traffic volumes equate to greater propagule pressure and climatic similarity results in a greater chance of exotic organism survival. For species-specific studies, this generic relationship between climatic similarity and traffic volume on a route could be adapted to match prior knowledge on the tolerances and preferences of the species.

While CED and CED*t* identify potential high-risk routes within the WAN for dispersal and establishment of climatically sensitive biota, we also aim to identify those airports with multiple scheduled links to climatically similar regions. The ‘climatic similarity index’ (CSI) of an airport is therefore proposed as a measure of this. The CSI of an airport *i* is defined as

$${\text{CSI}}_{i}=\frac{d_{i}}{\sum {\text{CED}}_{ij}},$$

where CED_*ij*_ is the CED between airports *i* and *j*. The degree *d*_*i*_ of airport *i* within the WAN is defined as the number of other airports to which it is connected by incoming direct scheduled routes. The CSI of an airport is low if it is connected by scheduled routes to very few other airports, or if it is connected to many other airports, but the climates at these airports are, on average, very different from that at the airport in question. The CSI of airport *i* is high if it is connected to many other airports where, on average, the climate is very similar to that at airport *i* (figure 2 in the electronic supplementary material displays the CSI features of the WAN).

Airports with relatively high CSIs are at increased invasion risk if incoming traffic levels are also high. The effects of both traffic volumes and climatic similarity on individual airports were examined by calculating, for each airport, the traffic-scaled CSI, CSI*t*. The traffic-scaled CSI of airport *i*, CSI*t*_*i*_, is defined as

$$\text{CSI}t_{i}={\text{CSI}}_{i}\left(\frac{\sum t_{j\to i}}{t{\left(m\right)}_{\text{max}}}\right),$$

where *t*_*j*→*i*_ is the total monthly seat capacity incoming to airport *i* from airport *j*, and *t*(*m*)_max_ is the global maximum total seat capacity for month *m*. Thus, the airports with the highest CSI*t* values are those with numerous high traffic volume incoming routes from climatically similar regions.

## 3. Results

### (a) Large-scale climatic connectivity structure of the worldwide airline transportation network

Figure 1 demonstrates that the average CED of all the routes of the WAN under study is lowest in the June–August period. The plot shows that the current structure of the WAN results in seasonal variation in its climatic similarity, with lowest overall levels (i.e., highest CEDs) in December and highest levels of similarity (i.e., lowest CEDs) in June. This suggests that climatically sensitive organisms moving on the WAN are, overall, more likely to find their arrival destinations habitable from June to August. Importantly, figure 1 shows that this June–August climatic similarity peak coincides with peak traffic volumes within the WAN. Thus, globally, not only is the WAN more conducive to biological invasion through its climatic similarity in June–August, but also increased propagule pressure at this time compounds that risk.

### (b) Climatic similarity on individual routes

Within the WAN, there exist numerous routes that link spatially distant, but climatically very similar regions. It is such routes that can promote biological exchange between areas where separation has resulted in differing species assemblages adapted to the same climate. For each month, CEDs between the origin and destination airports were binned into ten equal-interval categories. Figure 2*a* shows the relationship between these classes and the length of each route for July (plots were of similar shape for all months). As one would expect, in general, the further two airports are apart spatially, the more dissimilar their climates.

For each CED class, outliers exist which do not conform to the general rule of climatic spatial dependence. These represent one of two situations: (i) routes connecting airports which are spatially very close, but climatically dissimilar due to local climatic phenomena and (ii) routes connecting airports which are spatially distant, but climatically very similar. It is this second type of route that is potentially more vulnerable to exotic biological exchange. Ideally, identification of the most vulnerable routes should be based on the greatest differences in biogeographic history rather than spatial distance, however, in the absence of globally consistent fine resolution data on this, we assume that distance adequately captures such information. Such outlier routes are plotted and highlighted on figure 2*b* for July and table 1 in the electronic supplementary material lists the 20 routes in the lowest CED class separated by the greatest geographical distances for January, April, July and October by length from the 5% outliers (figure 3 in the electronic supplementary material shows all the 5% outlier routes). A significantly greater number of long distance routes is seen in July than January, many of which provide a Northern–Southern Hemisphere connection, with April and October showing intermediate levels. Climatic similarity between the northern Europe, eastern USA and Canadian summers are evident in the July list of routes, as are Iberia–South America and Japan–Pacific Island routes, with other long haul routes falling just outside the top 20 listed, for example, Frankfurt–Kilimanjaro. The other three periods studied show more Far East to Europe or North America routes, as climatic similarity between these regions peaks. The CEDs throughout the year for the four routes at the top of table 1 in the electronic supplementary material are shown in figure 4 in the electronic supplementary material.

### (c) Subnetworks

The WAN has important subnetworks of climatically similar airports. Figure 3 maps those subnetworks that encompass more than 10 airports or airports from three or more countries, and shows that subnetworks of climatic similarity spanning the globe are evident throughout the year. For January, the largest group encompasses the majority of eastern USA airports, many Mediterranean and some central and northeastern Asian airports, while another large subnetwork links the majority of European airports with those in Japan and eastern China. These specific linkages of spatially distant airports do not persist throughout the year (figures 3*b*–*d*) as climates diverge. In April (figure 3*b*), more spatially contiguous subnetworks develop, though exceptions are seen, with west coast US airports alongside Middle Eastern and eastern South African airports (pink circles). Such a spatially dispersed subnetwork, incorporating airports in north, south, east and west hemispheres, is not seen at other times of the year. Subnetworks encompassing some significant Northern–Southern Hemisphere links are evident in July (figure 3*c*), one of the months of greatest climatic homogeneity in the WAN. During this period, a large number of European airports appear in the same subnetwork as eastern coast South American airports, while inland South American airports are linked with those in Iberia and North Africa. As well as these north–south links, east–west subnetworks are evident, grouping airports in (i) eastern USA and southern Europe, (ii) Hawaii and east Asia, (iii) South America, southwest Africa, Australia and New Zealand. As climatic similarity in the WAN shifts again in October, further seasonally characteristic and significant subnetworks appear. Spatially diverse subnetworks are seen to incorporate airports in (i) Hawaii, Central America and the Caribbean, (ii) southern Europe and South America again, (iii) China, Japan and eastern Australia, (iv) West Africa and northeast Brazil.

### (d) Traffic

June stands out more clearly as the month with the highest CED*t* values than it did as the month with the lowest CED values, suggesting that, in general, the scheduled flight routes with the largest traffic capacities connect cities which are most climatically similar in June. Thus, the increased risk of successful biological invasion through greater propagule pressure on certain routes is exacerbated in the Northern Hemisphere summer period by these routes connecting climatically similar regions.

Table 2 in the electronic supplementary material lists the 30 airports within the international WAN with the highest CSI*t* values averaged across the 12 months of the year. There is some broad correspondence with the top airports globally in terms of total incoming traffic (table 3 in the electronic supplementary material) and both are dominated by US airports due to the busy US domestic network. Figure 4*a* plots those airports where the difference in ranking is 25 places or greater. Many airports show a global CSI*t* ranking that is considerably higher than their global incoming traffic ranking (orange to red colours). This indicates that the routes with the highest traffic volumes incoming to such airports are originating in climatically similar regions. In contrast, others have CSI*t* rankings that are considerably lower than their standings in terms of incoming traffic volumes (yellow colours), indicating that the major high volume routes operating into these airports originate in climatically dissimilar regions. In terms of relative biological invasion risk, therefore, those airports with higher CSI*t* than traffic rankings are predicted to be at greater risk. Figure 4*a* demonstrates that such airports are often grouped spatially, with ‘hot spots’ occurring in central–north USA, the Caribbean, central–west Europe, southern Scandinavia and eastern Asia.

Figure 4*b* shows the CSI*t* values of each airport in January and figure 4*c* shows the change in CSI*t* from the January baseline for each airport in July. Airports where CSI*t* has increased from January to July (red colours, figure 4*c*) indicates increased levels of traffic incoming from airports where the climate is converging in similarity with the airport in question and thus, an increased risk of climatically sensitive biota invasion. Figure 4*c* exhibits spatially distinct patterns, with CSI*t* decreasing in July across equatorial regions, central Europe, southern Australia, central and northern North America and southern South America. Elsewhere, CSI*t* increases are evident. Variations, however, are evident and often not spatially dependent, reflecting the variety of scheduled international routes operated from neighbouring airports.

## 4. Discussion

The evolution of the WAN has enabled some of the world's most diverse and isolated ecosystems to become connected via high speed transport and accelerate biological invasions. The initial dispersal phase of biological invasion, upon which the other stages rely, remains the least studied aspect of ‘invasion’ research (Puth & Post 2005). This is puzzling, given the importance of this phase, the proven link between human transport and biological invasion, and since a major aspect to the initial dispersal phase, propagule pressure, remains the strongest overall predictor of invasion success (Kolar & Lodge 2001; Levine & D'Antonio 2003; Drake & Lodge 2004; Lockwood *et al*. 2005). Though countless other factors have been put forward as predictors of species invasion success (Gewin 2005), we have focused here on providing a baseline analysis of those factors shown to be important to the initial dispersal and survival phases at a global scale (Tatem *et al*. 2006*a*–*c*).

The results outlined in this paper show that the current architecture of the WAN provides links between regions of high climatic similarity, but that are spatially distant and, therefore, exhibiting differing biogeographic histories. These linkages change seasonally, showing greatest overall climatic similarity in June, July and August, when long haul routes link climatically similar regions around the globe. This above average connection of similar climates raises the overall chances of successful biological invasion by climatically sensitive biota through the WAN. The coincident peak in air traffic volumes and consequent higher propagule pressure further increases these risks. The range of destinations from which incoming flights arrive at each airport means that there exists considerable local variation in incoming CEDs and airport CSI*t* values, even between neighbouring airports. Quantifying when, how and where heightened biological invasion risks may occur enables surveillance and controls authorities to target their efforts.

Examination of important subnetworks within the climatically weighted WAN reveals seasonal and locally diverse patterns of climatic similarity. Figure 3*a* shows that central and western Europe are climatically most similar to Japan and China (the fastest growing air freight exporter (IATA 2006)) in January, but to the eastern USA six months later. As a further example, Hawaii has one of the most studied and invaded ecosystems (Loope 1998) and figure 3*b*–*d* demonstrates similarly, how its climatically closest air links change from central America in April, to Asia in July, then the Caribbean in October. Figure 4*a* also highlights how the highest incoming traffic volume routes originate in regions that are climatically similar to Hawaii. These high traffic incoming flight routes from a diverse range of destinations, working in synchrony with seasonal climatic shifts, show how favourable Hawaii is to incoming exotic biota from far and wide, and there exist many other airports with similar features.

The approaches and metrics outlined here point to applications and extension in a number of future directions. (i) The use of empirical evidence on specific climatic preferences and tolerances, and current known distributions of potential invasive species, incorporated into the analyses described here, can be used to highlight per species movement risk routes in the WAN ([Bibr bib25][Bibr bib26][Bibr bib27][Bibr bib28]). For invasive organisms with the ability to remain dormant through unfavourable climatic conditions, this may include analysis of phase-shifts to identify routes connecting locations where, for example, CEDs are nearly identical if conditions at one location are shifted forward a few months. (ii) The adoption of a probabilistic context, enabling organism introduction to the WAN to be treated as a stochastic process and flights as independent organism dispersal events. (iii) Extension of the analyses described here to seaborne trade, land transport and transport hub catchment areas, would form an integrated approach to climatic similarity analysis of global human transport networks, enabling refinement of inspection and fumigation strategies. (iv) Combining climate change predictions with future travel projections allows estimates of WAN climatic similarity changes to be made (Tatem *et al*. 2006*d*). (v) The findings here could potentially be incorporated as parameters into modelling of climate-sensitive infectious disease spread (Cazalles & Hales 2006), for example, influenza (Viboud *et al*. 2006).

The rapid expansion of the WAN means that organisms have never had a better chance at expanding their ranges. Those that have moved just once or twice in the past now have countless opportunities to do so again via the WAN. Combined with expected increases in global trade and travel, particularly through the WAN, the environmental and economic impacts of these invaders are not likely to diminish. Our best hope of minimizing these impacts remains through the optimization of prediction, surveillance and control at international transport gateways.

## Figures and Tables

**Figure 1:**
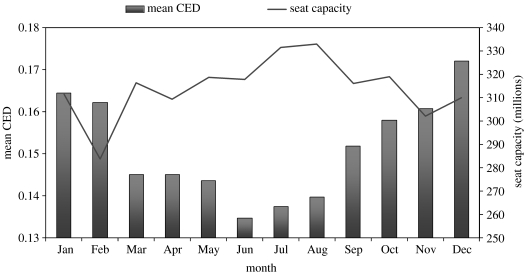
2005–2006. Mean monthly CEDs and monthly total seat capacities across the WAN.

**Figure 2:**
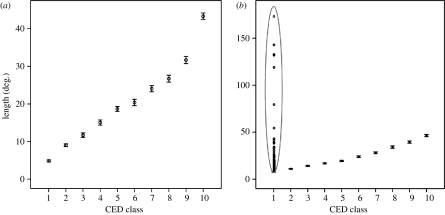
(*a*) Plot of CEDs, binned into 10 equal classes (approx. 4000 routes in each class), against route length (bars show 95% range); (*b*) the same plot with the 5% positive outlier airports in class 1 plotted and highlighted.

**Figure 3:**
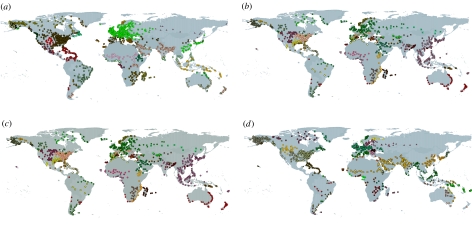
Subnetworks (encompassing more than 10 airports or more than three countries) within the CED-weighted WAN for (*a*) January, (*b*) April, (*c*) July and (*d*) October (those airports with the same colour and style of icon represent a subnetwork).

**Figure 4:**
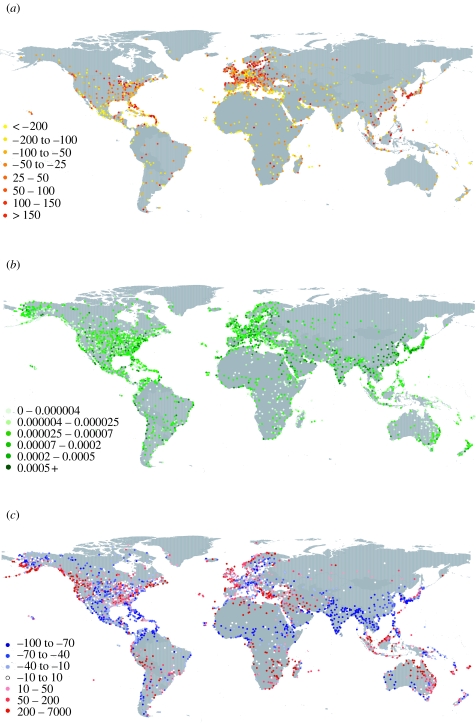
(*a*) Difference between annual average traffic-scaled climatic similarity index (CSI*t*) ranking and traffic (*t*) ranking, calculated by rank(CSI*t*)-rank(*t*). Those airports with a ranking difference of less than 25 places were not shown. (*b*) CSI*t* distribution for January; (*c*) percentage change in CSI*t* from January to July.

## Supplementary Material

Tatem—electronic supplementary material Electronic supplementary figures and tables
