# Supplementary material for: Climatic similarity and biological exchange in the worldwide airline transportation network
Source: Proc Biol Sci. 2007 Apr 10;274(1617):1489–96. doi: 10.1098/rspb.2007.0148 (PMC1914332; doi:10.1098/rspb.2007.0148)
Supplement: Supplementary file 1 [file rspb20070148s02.doc]

Figure 1. Plots of total monthly (a) seat capacity, (b) hold volume and (c) tonnage, across the WAN.

Figure 2. Mean monthly *CSI* across the WAN.

(a) (b)

(b) (d)

Figure 3. The complete set of 5% outlier routes mapped for (a) January, (b) April, (c) July and (d) October.

(a)	(b)

(c)	(d)

Figure 4. Plots of monthly climatic Euclidean distances between (a) Chicago and Seoul, (b) Los Angeles and Tel Aviv, (c) Buenos aires and Sydney and (d) Philadelphia and Seoul.

| **January** | **April** | **July** | **October** |
| --- | --- | --- | --- |
| Chicago, *USA* – Seoul, *Republic of Korea* | Los Angeles, *USA* – Tel Aviv, *Israel* | Buenos Aires, *Argentina* – Sydney, *Australia* | Philadelphia, *USA* – Seoul, *Republic of Korea* |
| San Francisco, *USA* – Rome, *Italy* | Chicago, *USA* – Seoul, *Republic of Korea* | San Francisco, *USA* – Frankfurt, *Germany* | Montevideo, *Uruguay* – Barcelona, *Spain* |
| Jeddah, *Saudi Arabia* – Manila, *Philippines* | San Francisco, *USA* – Rome, *Italy* | Vancouver, *Canada* – Dusseldorf, *Germany* | Seoul, *Republic of Korea* – Christchurch, *New Zealand* |
| Dakar, *Senegal* – Jeddah, *Saudi Arabia* | Seattle, *USA* – Frankfurt, *Germany* | Vancouver, *Canada* – London, *UK* | Toronto, *Canada* – Munich, *Germany* |
| Milan, *Italy* – Tehran, *Iran* | Toronto, *Canada* – Munich, *Germany* | Toronto, *Canada* – Budapest, *Hungary* | New York, *USA* – Vence, *Italy* |
| Jeddah, *Saudi Arabia* – Chennai, *India* | Montreal, *Canada* – Munich, *Germany* | Montreal, *Canada* – Vienna, *Austria* | Montreal, *Canada* – Munich, *Germany* |
| Lome, *Togo* – Jeddah, *Saudi Arabia* | New York, *USA* – Milan, *Italy* | Toronto, *Canada* – Milan, *Italy* | New York, *USA* – Bologne, *Italy* |
| Dubai, *UAE* – Chittagong, *Bangladesh* | New York, *USA* – Zurich, *Switzerland* | Rio de Janeiro, *Brazil* – Paris, *France* | Montevideo, *Uruguay* – New York, *USA* |
| Niamey, *Niger* – Khartoum, *Sudan* | San Francisco, *USA* – Melbourne, *Australia* | Toronto, *Canada* – Lyon, *France* | Sal, *Cape Verde* – Luanda, *Angola* |
| Oslo, *Norway* – Moscow, *Russia* | Vancouver, *Canada* – New York, *USA* | Boston, *USA* – Venice, *Italy* | Casablanca, *Morocco* – Beirut, *Lebanon* |
| Rome, *Italy* – Amman, *Jordan* | Santa Cruz, *Bolivia* – Havana, *Cuba* | Boston, *USA* – Rome, *Italy* | Frankfurt, *Germany* – Krasnodar, *Russia* |
| Rome, *Italy* – Aleppo, *Syria* | Casablanca, *Morocco* – Beirut, *Lebanon* | Toronto, *Canada* – Toulouse, *France* | Niamey, *Niger* – Khartoum, *Sudan* |
| Freetown, *Sierra Leone* – Douala, *Cameroon* | Edinburgh, *UK* – Moscow, *Russia* | New York, *USA* – Barcelona, *Spain* | San Jose, *USA* – Mexico City, *Mexico* |
| Karachi, *Pakistan* – Dhaka, *Bangladesh* | London, *UK* – Moscow, *Russia* | Caracas, *Venezuela* – Barcelona, *Spain* | Melbourne, *Australia* – Christchurch, *New Zealand* |
| Lima, *Peru* – San Jose, *Costa Rica* | Dusseldorf, *Germany* – Trabzon, *Turkey* | Halifax, *Canada* – Munich, *Germany* | Kuwait, *Kuwait* – Lahore, *Pakistan* |
| Casablanca, *Morocco* – Catania, *Italy* | Cologne, *Germany* – Trabzon, *Turkey* | Rio de Janeiro, *Brazil* – Porto, *Portugal* | Munich, *Germany* – Donetsk, *Ukraine* |
| Rome, *Italy* – Larnaca, *Cyprus* | Amsterdam, *Netherlands* – Moscow, *Russia* | Halifax, *Canada* – Frankfurt, *Germany* | Fortaleza, *Brazil* – Sal, *Cape Verde* |
| Edmonton, *Canada* – Minneapolis/St Paul, *USA* | Frankfurt, *Germany* – Rostov, *Russia* | Tokyo, *Japan* – Nadi, *Fiji* | Kuwait, *Kuwait* – Ahmedabad, *India* |
| Nice, *France* – Istanbul, *Turkey* | London, *UK* – Kiev, *Ukraine* | Honolulu, *USA* – Nagoya, *Japan* | Yerevan, *Armenia* – Tashkent, *Uzbekistan* |
| Casablanca, *Morocco* – Tripoli, *Libya* | Melbourne, *Australia* – Palmerston, *New Zealand* | Sao Paulo, *Brazil* – Madrid, *Spain* | Al Ain, *UAE* – Amman, *Jordan* |

Table 1. The 20 longest distance scheduled routes within CED class 1.

| **Rank** | **Airport** | **Mean CSI*t*** |
| --- | --- | --- |
| 1 | Chicago ORD, *USA* | 0.0208 |
| 2 | Atlanta *USA* | 0.0198 |
| 3 | London LHR, *UK* | 0.0160 |
| 4 | Los Angeles, *USA* | 0.0154 |
| 5 | Dallas/Fort Worth, *USA* | 0.0150 |
| 6 | Paris CDG, *France* | 0.0131 |
| 7 | Denver, *USA* | 0.0129 |
| 8 | Frankfurt, *Germany* | 0.0120 |
| 9 | Detroit, *USA* | 0.0114 |
| 10 | Philadelphia, *USA* | 0.0114 |
| 11 | Madrid, *Spain* | 0.0108 |
| 12 | Tokyo NRT, *Japan* | 0.0104 |
| 13 | Barcelona, *Spain* | 0.0101 |
| 14 | Amsterdam, *Netherlands* | 0.0101 |
| 15 | Minneapolis/St Paul, *USA* | 0.0100 |
| 16 | Phoenix, *USA* | 0.0095 |
| 17 | Houston, *USA* | 0.0094 |
| 18 | Toronto, *Canada* | 0.0091 |
| 19 | Orlando, *USA* | 0.0090 |
| 20 | Boston, *USA* | 0.0088 |
| 21 | Baltimore, *USA* | 0.0088 |
| 22 | New York JFK, *USA* | 0.0088 |
| 23 | Charlotte, *USA* | 0.0087 |
| 24 | Las Vegas, *USA* | 0.0085 |
| 25 | New York LGA, *USA* | 0.0085 |
| 26 | Copenhagen, *Denmark* | 0.0077 |
| 27 | Beijing, *China* | 0.0075 |
| 28 | Cincinnati, *USA* | 0.0071 |
| 29 | Stockholm, *Sweden* | 0.0069 |
| 30 | New York EWR, *USA* | 0.0069 |

Table 2. Top 30 airports by mean annual CSI*t*.

| RANK | CITY | COUNTRY | TRAFFIC-SUM |
| --- | --- | --- | --- |
| 1 | LOS ANGELES | UNITED STATES OF AMERICA | 72764774 |
| 2 | ATLANTA | UNITED STATES OF AMERICA | 67409908 |
| 3 | CHICAGO | UNITED STATES OF AMERICA | 64693605 |
| 4 | LONDON | UNITED KINGDOM | 58419495 |
| 5 | DALLAS/FORT WORTH | UNITED STATES OF AMERICA | 48004663 |
| 6 | PARIS | FRANCE | 45583520 |
| 7 | HONG KONG | HONG KONG (SAR) CHINA | 44922375 |
| 8 | LAS VEGAS | UNITED STATES OF AMERICA | 42811825 |
| 9 | DENVER | UNITED STATES OF AMERICA | 41305935 |
| 10 | PHOENIX | UNITED STATES OF AMERICA | 40987382 |
| 11 | FRANKFURT | GERMANY | 40761920 |
| 12 | TAIPEI | CHINESE TAIPEI | 40511804 |
| 13 | TOKYO | JAPAN | 38714714 |
| 14 | BEIJING | CHINA | 36862673 |
| 15 | MADRID | SPAIN | 35970961 |
| 16 | BANGKOK | THAILAND | 35888965 |
| 17 | HOUSTON | UNITED STATES OF AMERICA | 34643510 |
| 18 | NEW YORK | UNITED STATES OF AMERICA | 34398414 |
| 19 | SYDNEY | AUSTRALIA | 33985510 |
| 20 | ORLANDO | UNITED STATES OF AMERICA | 31576757 |
| 21 | AMSTERDAM | NETHERLANDS | 31513680 |
| 22 | PHILADELPHIA | UNITED STATES OF AMERICA | 31446777 |
| 23 | SEATTLE | UNITED STATES OF AMERICA | 30818245 |
| 24 | DETROIT | UNITED STATES OF AMERICA | 30319614 |
| 25 | MINNEAPOLIS/ST PAUL | UNITED STATES OF AMERICA | 29486568 |
| 26 | SINGAPORE | SINGAPORE | 29331779 |
| 27 | SEOUL | KOREA REPUBLIC OF | 28779616 |
| 28 | MIAMI | UNITED STATES OF AMERICA | 28756996 |
| 29 | TOKYO | JAPAN | 28545072 |
| 30 | TORONTO | CANADA | 27673537 |

Table 3 Top 30 airports by incoming traffic, as defined by total annual incoming seat capacity.
